# Supplementary material for: UHPLC-ESI-OT-MS Phenolics Profiling, Free Radical Scavenging, Antibacterial and Nematicidal Activities of “Yellow-Brown Resins” from Larrea spp
Source: Antioxidants (Basel). 2021 Jan 28;10(2):185. doi: 10.3390/antiox10020185 (PMC7911333; doi:10.3390/antiox10020185)
Supplement: Supplementary file 1 [file antioxidants-10-00185-s001.pdf]

antioxidants

UHPLC-ESI-OT-MS phenolics profiling, free radical scavenging, antibacterial and nematicidal activities of "yellow-brown resins" from Larrea spp.

Jessica Gómez[1,2], Mario J. Simirgiotis[3,4,*], Sofía Manrique[1,2], Mauricio Piñeiro[1,2], Beatriz Lima[1,2], Jorge Bórquez[5] and Gabriela E. Feresin[1,2]and Alejandro Tapia[1*]

1 Instituto de Biotecnología-Instituto de CienciasBásicas, Universidad Nacional de San Juan,Av. Libertador General San Martín 1109 (O), San Juan CP 5400, Argentina; jesicagomez674@gmail.com (J.G.); manriquesofia2@gmail.com (S.M.); mauridpg@gmail.com(M.P.); blima@unsj.edu.ar(B.L.); gferesin@unsj.edu.ar(G.E.F.)
2 CONICET (Consejo Nacional de Ciencia y Tecnología), CABA, Buenos Aires C1405DJR, Argentina
3 Instituto de Farmacia, Facultad de Ciencias, Campus Isla Teja, Universidad Austral de Chile, Valdivia 5090000, Chile
4 Center for Interdisciplinary Studies on the Nervous System (CISNe), Universidad Austral de Chile, Valdivia 5090000, Chile
5 Laboratorio de Productos Naturales Depto. de Química, Facultad de Ciencias, Universidad de Antofagasta, Av. Coloso S-N, Antofagasta 1240000, Chile; jorge.borquez@uantof.cl
* Correspondence: mario.simirgiotis@gmail.com (M.J.S.); atapia@unsj.edu.ar (A.T.); Tel.: +56-063-2244369 (M.J.S.); +54-264-4211700-294 (A.T.)

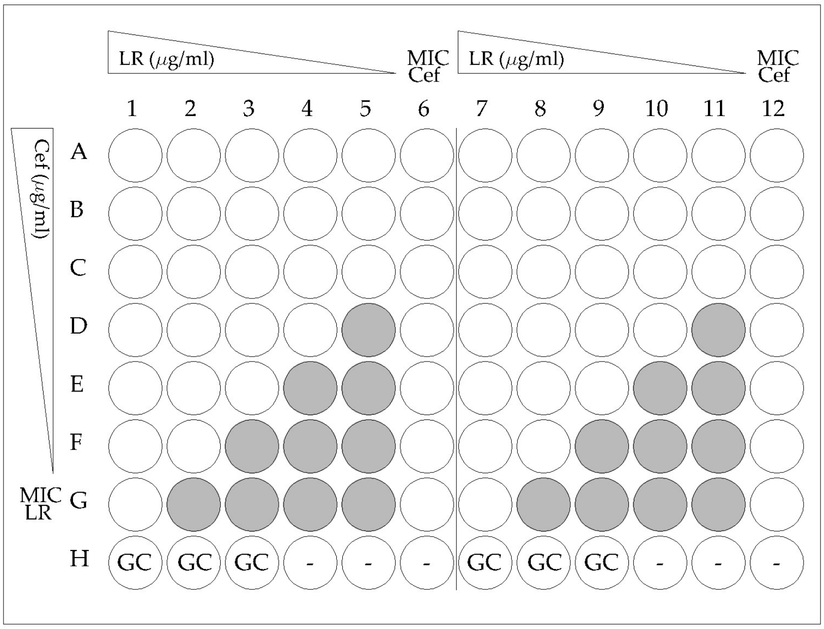

Figure S1. Representative panel of Checkerboard design combining Larrea resins (LR) with Cefotaxime (Cef). GC stands for growth control. Dark circles show bacterial growth and white circles show no bacterial growth.

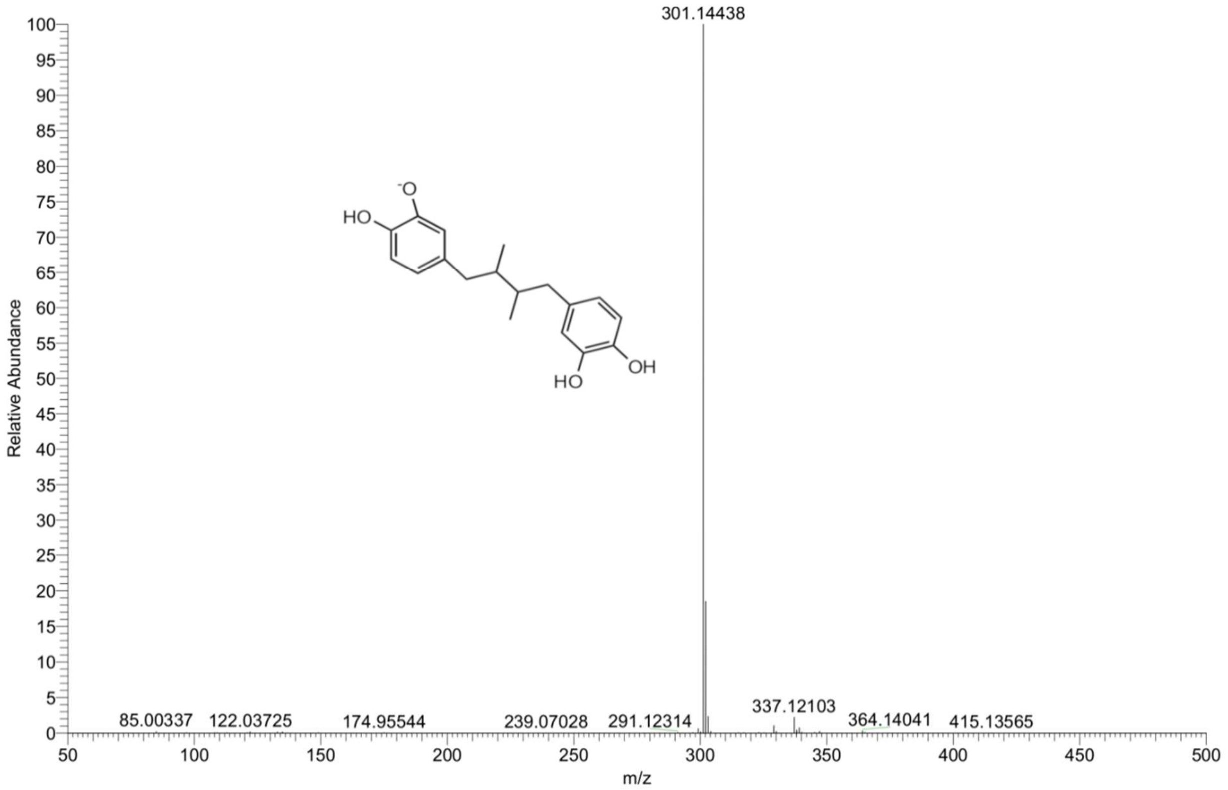

Figure 2. Full orbitrap MS spectra and structures of representative compounds 13, 16, 20, 26 and 28 (a-e). a**.** Compound 13.

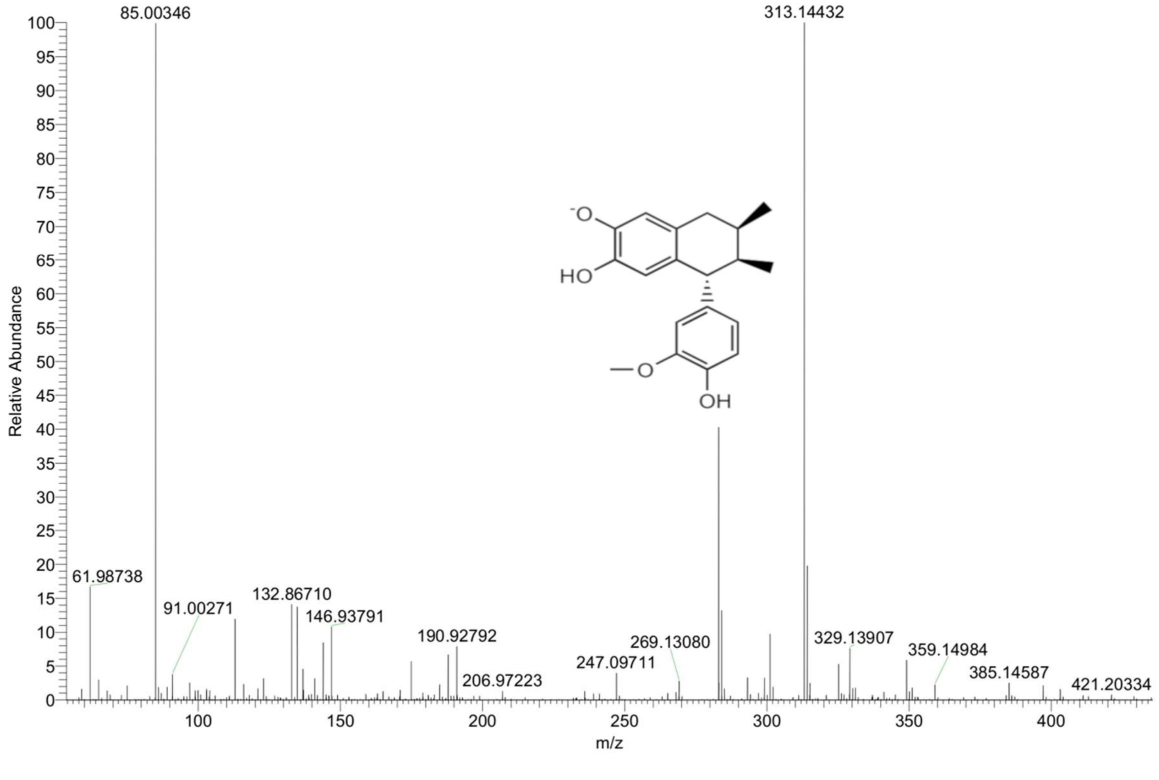

Figure 2. b.Compound 16.

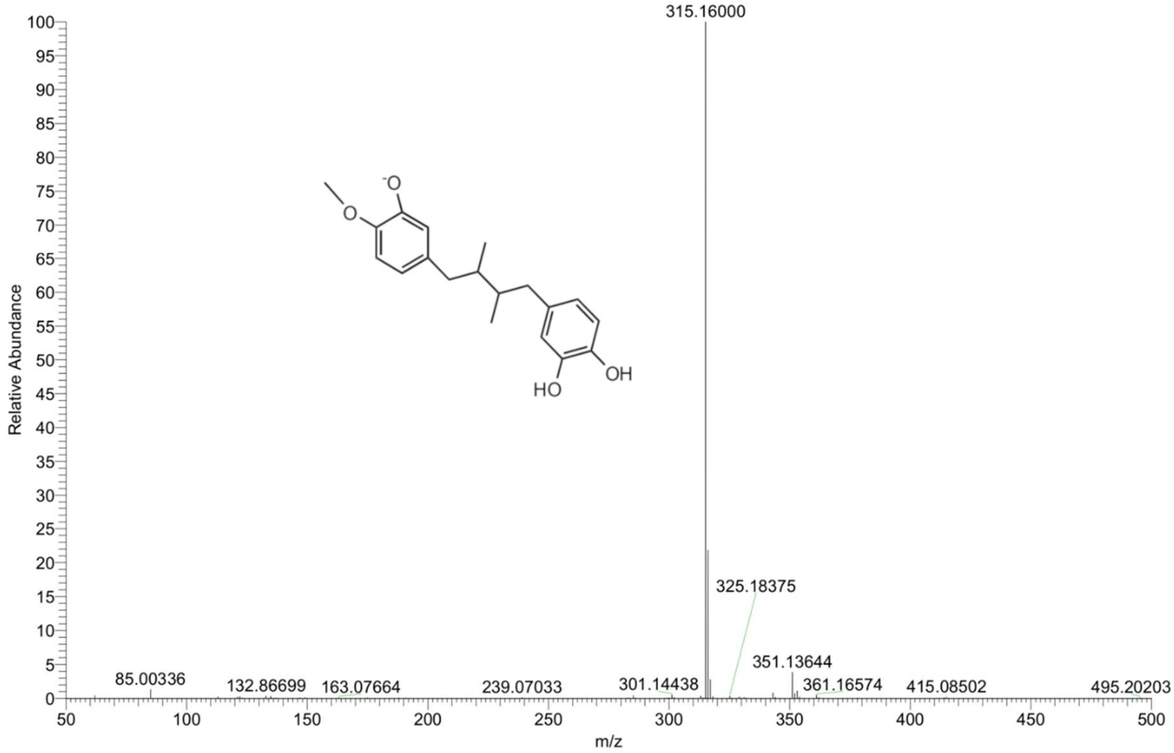

Figure 2. c. Compound 20.

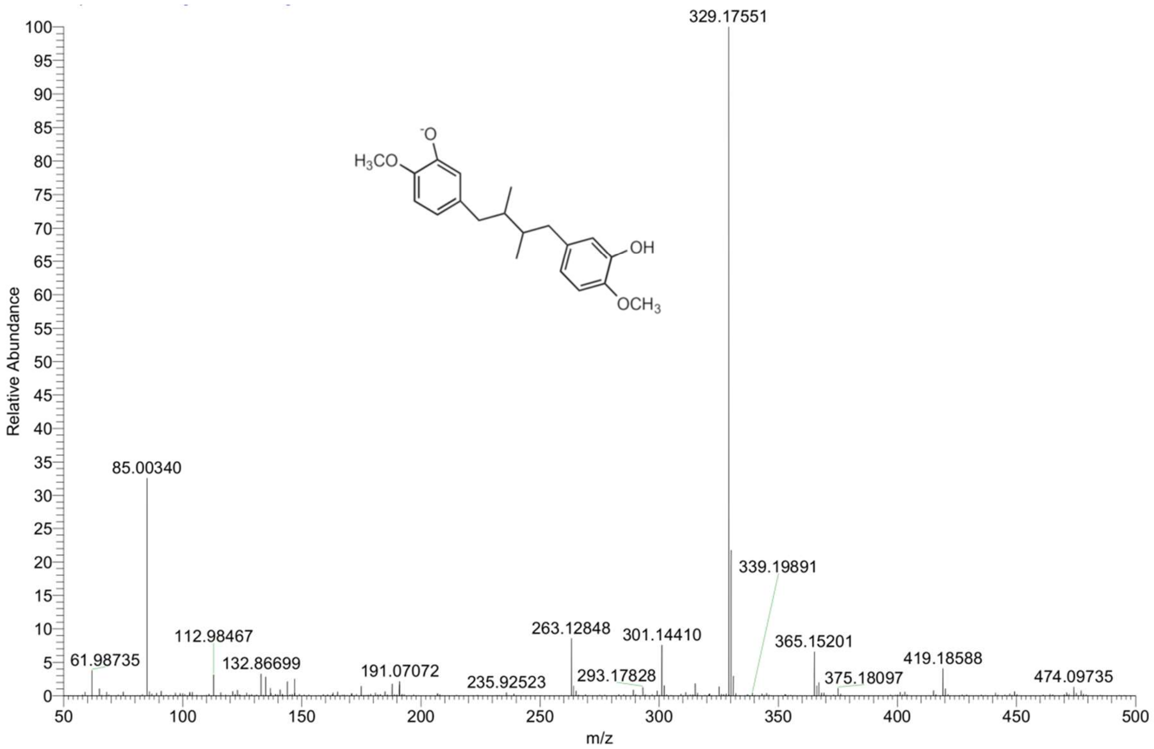

Figure 2. d. Compound 26.

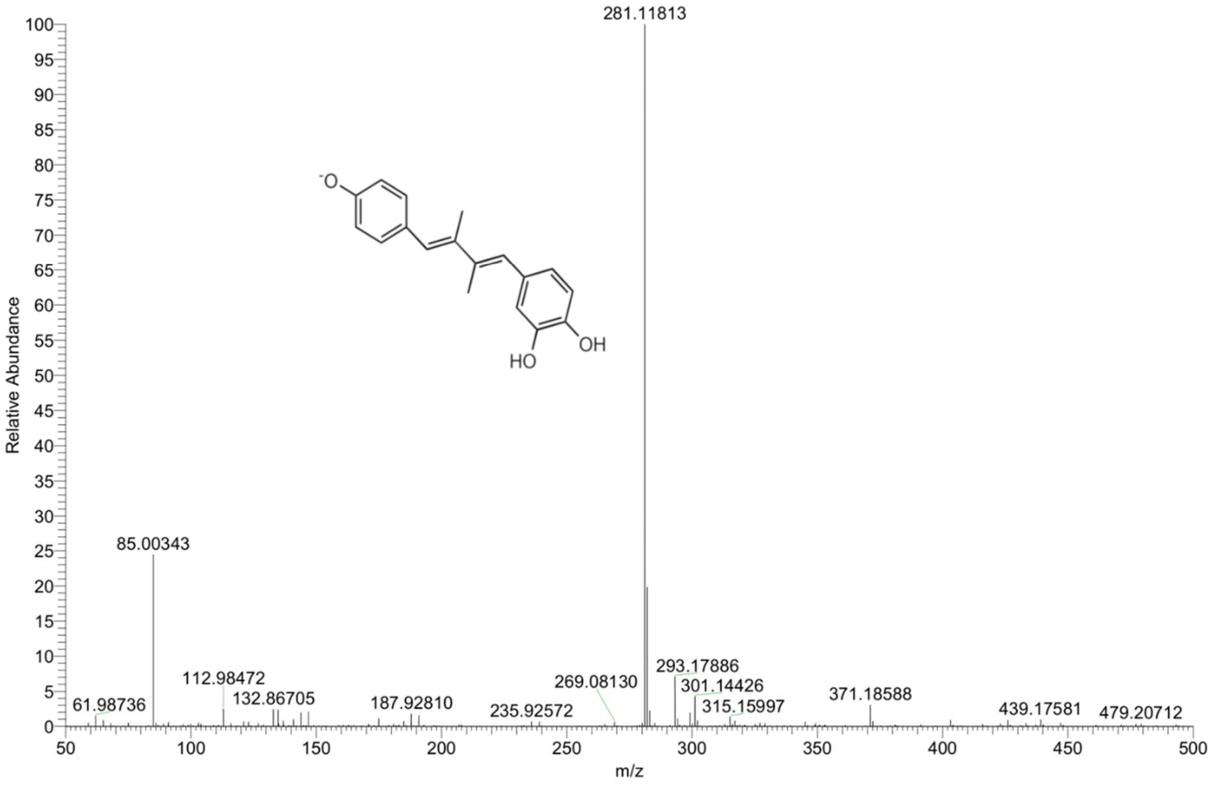

Figure 2. e. Compound 28.
